# EVALUATING THE VALIDITY AND QUALITY OF SELF-REPORTED IMPORTANT LIFE ACCOMPLISHMENTS BY ADULTS OVER AGE 50

**DOI:** 10.1093/geroni/igae098.2854

**Published:** 2024-12-31

**Authors:** Wenshan Yu, Jacqui Smith

**Affiliations:** Duke University, Ann Arbor, Michigan, United States; University of Michigan, Ann Arbor, Michigan, United States

## Abstract

Bluck (2003) proposed that autobiographical memories function to maintain a sense of fulfillment and well-being. In support of this, studies find that older adults prioritize reporting positive early-life content in life narratives. Questions remain however, about the validity of life narratives and whether content prioritization is observed in non-narrative contexts. We utilized data obtained in the Health and Retirement Study (HRS) Life History Mail Survey (LHMS: N = 10496, M age = 69.5) to ask if the content of participants’ narrative reports about personal life accomplishments was associated with the quality (item of missing rates; IMR) of responses to non-narrative retrospective questions about residential, education, job, marriage, caregiving, and health histories. Content analyses of the life accomplishment narratives revealed 16 topics relevant to autobiographical life history content (interrater reliability=0.921). Compared to HRS participants who did not provide narrative life accomplishments, those who did had higher cognitive status and more years of education (cognitive status OR=1.04, p< 0.001; education OR=1.02, p< 0.001). Multinomial regressions indicated gender-related narrative content differences: whereas men reported career-related accomplishments, women prioritized accomplishments such as children, marriage, and pride in self (OR=6.17 p< 0.001). Linear regressions revealed that when participants mentioned corresponding accomplishments (i.e., about education, career, care for others, health, and marriage), their IMRs significantly decreased in that section, even after controlling for demographic, socioeconomic, and health variables (p < 0.01). This suggests that the validity and quality of life history data is influenced by participants’ attitudes and beliefs about life in addition to their sociodemographic characteristics.

